# *Burkholderia pseudomallei* OMVs derived from infection mimicking conditions elicit similar protection to a live-attenuated vaccine

**DOI:** 10.1038/s41541-021-00281-z

**Published:** 2021-01-29

**Authors:** Sarah M. Baker, Erik W. Settles, Christopher Davitt, Patrick Gellings, Nicole Kikendall, Joseph Hoffmann, Yihui Wang, Jacob Bitoun, Kasi-Russell Lodrigue, Jason W. Sahl, Paul Keim, Chad Roy, James McLachlan, Lisa A. Morici

**Affiliations:** 1 Department of Microbiology and Immunology, School of Medicine, Tulane University, New Orleans, LA USA; 2 Pathogen & Microbiome Institute, Northern Arizona University, Flagstaff, AZ USA; 3 Tulane National Primate Research Center, Covington, LA USA

**Keywords:** Infectious diseases, Vaccines, Vaccines

## Abstract

*Burkholderia pseudomallei* is a Gram-negative, facultative intracellular bacillus that causes the disease melioidosis. *B. pseudomallei* expresses a number of proteins that contribute to its intracellular survival in the mammalian host. We previously demonstrated that immunization with OMVs derived from *B. pseudomallei* grown in nutrient-rich media protects mice against lethal disease. Here, we evaluated if OMVs derived from *B. pseudomallei* grown under macrophage-mimicking growth conditions could be enriched with intracellular-stage proteins in order to improve the vaccine. We show that OMVs produced in this manner (M9 OMVs) contain proteins associated with intracellular survival yet are non-toxic to living cells. Immunization of mice provides significant protection against pulmonary infection similar to that achieved with a live attenuated vaccine and is associated with increased IgG, CD4^+^, and CD8^+^ T cells. OMVs possess inherent adjuvanticity and drive DC activation and maturation. These results indicate that M9 OMVs constitute a new promising vaccine against melioidosis.

## Introduction

*Burkholderia pseudomallei* is a Gram-negative, facultative intracellular bacillus that causes the disease melioidosis^1^. Cases of human melioidosis have historically been most prevalent in Southeast Asia and Northern Australia, but newer modeling predicts that the disease is largely underestimated and is contributing to human morbidity and mortality in many tropical and sub-tropical regions of the world^2,3^. *B. pseudomallei* is inherently resistant to many broad-spectrum antibiotics, and antibiotic therapy regimens are often complicated and extensive. Furthermore, *B. pseudomallei* can establish a latent infection and relapse of disease can occur^4^. For these reasons, an effective prophylactic and/or therapeutic vaccine could help reduce the global public health burden caused by *B. pseudomallei*. In addition, *B. pseudomallei* is identified as a Tier 1 select agent with potential for biological misuse necessitating a vaccine for biodefense purposes as well.

Currently, there is no licensed vaccine available to prevent infection with *B. pseudomallei*, although numerous vaccine platforms have been evaluated in pre-clinical studies^5^. Our group previously showed that outer membrane vesicles (OMV) derived from *B. pseudomallei* are highly effective immunogens^6–9^. OMVs are naturally secreted by Gram-negative bacteria and serve numerous functions including, but not limited to, cell–cell communication, horizontal DNA transfer, predation, and delivery of virulence factors^10^. Several studies have demonstrated the protective efficacy of outer membrane vesicle-based vaccines^11,12^. We have shown that vaccination with *Burkholderia*-derived OMVs protects against multiple routes of infection and confers immunity to heterologous *B. pseudomallei* challenge in mice and *B. mallei* in mice and rhesus macaques^6,7,9^. However, the OMV vaccine-mediated protection was incomplete since some vaccinated mice succumbed to infection and bacteria were able to persist in surviving animals. The OMV vaccines used in our previous work were produced from *B. pseudomallei* strain 1026b or *B. pseudomallei* strain Bp82 (ΔpurM, derived from strain 1026b^13^) grown in nutrient-rich Luria-Bertani (LB) broth. Preparation of OMVs in this manner resulted in a highly consistent, immunogenic, and non-toxic vaccine product as confirmed in both BALB/c mice and non-human primates^6–9^.

*B. pseudomallei*, like other facultative intracellular bacterial pathogens, possesses multiple, tightly regulated virulence determinants that contribute to its intracellular survival and persistence in the host^1,14^. For example, *B. pseudomallei* expresses proteins (i.e., SodC, AhpC) that counteract oxidative and nitrosative stress^15,16^, proteins that polymerize actin and mediate cell–cell spread (i.e., BimA, BimC)^17,18^, and proteins that suppress macrophage immune responses (i.e., TssM)^19^. *B. pseudomallei* also requires the Type three secretion system, T3SS-3, and the type six secretion system, T6SS-1, for survival in macrophages and virulence in the mammalian host^1^. Burtnick and Brett identified iron and zinc as the critical components that negatively regulate *B. pseudomallei* T3SS-3 and T6SS-1 expression in human macrophages. They demonstrated that *B. pseudomallei* grown in media depleted of zinc and iron express T3SS-3 and T6SS-1, whereas bacteria grown in media containing zinc and iron do not^20^. Based on these findings, we tested whether OMVs derived from *B. pseudomallei* grown in media depleted of zinc and iron (hereafter referred to as M9 OMV) could be enriched with intracellular stage-specific proteins, such as T3SS-3 or T6SS-1 proteins. We hypothesized that an OMV vaccine containing one or more intracellular virulence determinants may be more effective by directing the immune response towards antigens that are critical for bacterial survival in the host. In this regard, an enriched OMV vaccine would be more analogous to a live *B. pseudomallei* vaccine that can express intracellular stage-specific antigens upon immunization and cellular uptake.

Here we demonstrate that M9 OMVs contain a number of differentially expressed and intracellular stage specific proteins, including virulence determinants. We show that the non-replicating M9 OMV vaccine lacks any observable toxicity and provides similar or better immunogenicity and protection versus a live attenuated vaccine in a head to head comparator experimental challenge. Lastly, we demonstrate that OMVs are self-adjuvanting and drive dendritic cell maturation, activation, and cytokine secretion in vitro and in vivo. Collectively, these results indicate that M9 OMVs constitute a new and promising acellular vaccine platform for *B. pseudomallei*.

## Results

### OMVs derived from *B. pseudomallei* grown in M9 media are enriched with intracellular stage-specific proteins

**Fig. 1:**
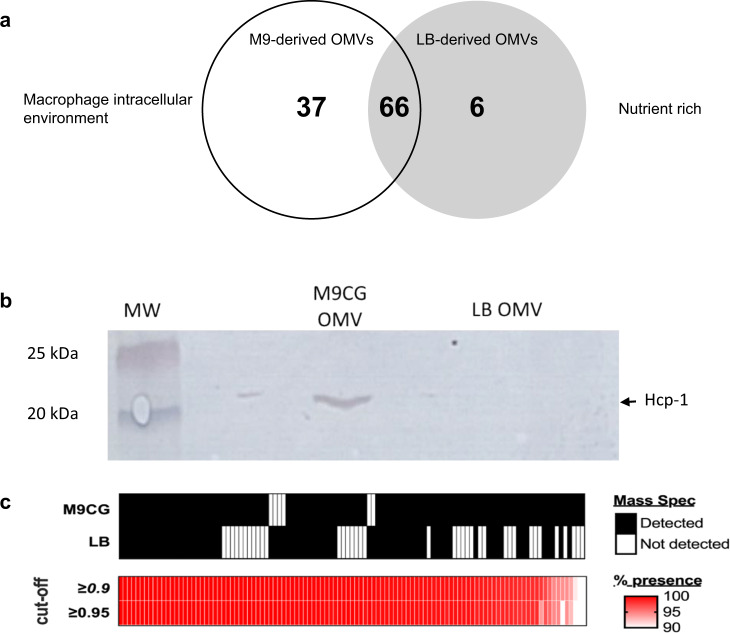
OMVs derived from *B. pseudomallei* grown in M9 media are enriched in intracellular-stage proteins. **a** Venn diagram depicting the unique and shared proteins found in OMVs derived from Bp grown in M9 versus LB media. 109 proteins are shown across the two preparations. **b** Presence of Hcp-1 was confirmed in M9 OMVs by Western blot with rat anti-Hcp-1 sera. Hcp-1 band is shown at 21 kDa. MW molecular ladder. **c** Large-scale blast score ratio (LS-BSR) for protein presence and conservation among *B. pseudomallei*. The conservation of proteins in the M9 and LB preparations was compared using tblastn LS-BSR analysis using coding regions from 407 sequenced *B. pseudomallei* genomes. Protein conservation was determined by blast score ratio cut-offs of ≥0.9 or ≥0.95. The percentage of the 407 tested genomes that contained each protein are shown in the heat map where <90% are shown in white and 100% are shown in red.

Changes in bacterial gene expression in response to environmental stimuli are often reflected in the composition of secreted outer membrane vesicles. We first sought to determine if proteins present in OMVs derived from Bp grown in M9 minimal media differed from OMVs obtained from Bp grown in LB media (LB OMV). Indeed, M9 OMVs contained 37 proteins that were distinct from LB OMV proteins and 66 proteins that were conserved in both preparations (Fig. 1a, Supplementary Table 1). Expression of the major component of the T6SS-1, hemolysin co-regulated protein (Hcp-1), is a reliable indicator of T6SS-1 expression by *B. pseudomallei*. The presence of Hcp-1 was observed exclusively in M9 OMVs as determined by LC/MS (Supplementary Table 1) and confirmed by Western blot (Fig. 1b). A number of other T6SS-1 proteins (e.g., tip protein VgrG; baseplate subunit TssK) and T3SS-3 proteins (e.g., effector protein BapC; guanine nucleotide exchange factor BopE) were also detected in M9, but not LB, OMVs by LC-MS (Supplementary Table 1). Additionally, M9 OMVs contained a number of lipoproteins (e.g., competence lipoprotein ComL), stress response proteins (e.g., Thioredoxin; alkylhydroperoxidase AhpD), and flagellar protein FlgG that were not detected in LB OMVs (Supplementary Table 1).

Next, the conservation and presence of the OMV proteins in the proteome of 407 *B. pseudomallei* isolates was determined. To do this, we performed a BLAST score ratio (BSR) analysis relative to 1026b, which is the parent isolate of Bp82. A BLAST bit score incorporates sequence identity and alignment length; a value of 1.0 is equivalent to 100% amino acid identity over 100% of the protein length in 1026b. Smaller values represent shorter alignments with lower sequence identity. Using a BSR value of ≥0.9, 86 out of 109 possible OMV proteins can be found in >99% of the *B. pseudomallei* isolates tested. If the BSR increased to ≥0.95, 84 out of 109 are still detected in >99% of the tested isolate genomes (Fig. 1c). We also examined conservation of proteins in the LB OMV preparation compared to the M9 OMV preparation. When a BSR cutoff of ≥90% was used, 63 of 66 M9 and LB common proteins, 32 of 37 M9 unique, and 6 of 6 LB unique proteins can be found in >95% of the *B. pseudomallei* isolates tested. Taken together, this data suggests that ~77% of the proteins detected in the *B. pseudomallei* OMV preparations are conserved in both amino acid identity and peptide length in a vast majority of *B. pseudomallei* isolates.

### M9 OMVs are non-toxic to living cells

**Fig. 2:**
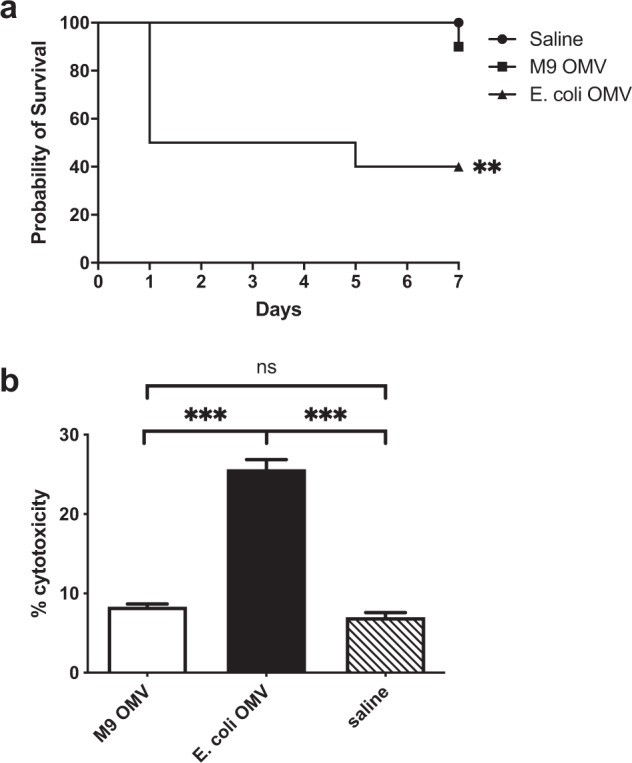
M9 OMVs are non-toxic to living cells. **a** *G. mellonella* (*n* = 10 per group) were inoculated with 10 μg Bp82 M9 OMVs, *E. coli* OMVs, or saline. *E. coli* OMVs caused significant mortality whereas M9 OMV and saline did not (**p < 0.01 by log rank Mantel Cox test). **b** RAW 264.7 murine macrophages were treated with saline, 10 μg M9 OMVs or *E. coli* OMVs and LDH release was measured 24 h later (***p < 0.001, n.s. = not significant by unpaired Student’s *t* test). Results shown are representative of three independent experiments.

Because M9 OMVs were enriched with proteins that contribute to bacterial virulence, we assessed whether they exhibited any toxicity towards living cells. First, we used a highly sensitive and well-established *Galleria mellonella* model for discrimination of OMV toxicity^21–23^. Larvae were inoculated with OMVs derived from Bp82 or a non-pathogenic strain of *E. coli* and monitored for 7 days. As seen in Fig. 2a, there was no significant difference in survival for saline- or M9 OMV-treated larvae, while OMVs derived from a non-virulent strain of *E. coli* caused significant (60%) mortality (*p* < 0.01). To confirm these results, we next treated RAW 264.7 macrophages with OMVs since antigen-presenting cells readily interact and phagocytose OMVs in vivo^24,25^. There was no cytotoxicity evident in M9 OMV treated cells compared to saline treated cells, whereas *E. coli*-derived OMVs did cause cytotoxicity (*p* < 0.001) (Fig. 2b). Together these results indicate that M9 OMVs are non-toxic to living cells, despite their selective enrichment with virulence proteins.

### Immunization with M9 OMVs provides similar protection to a live-attenuated vaccine

**Fig. 3:**
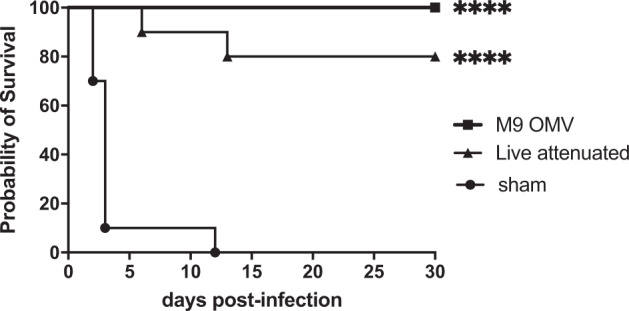
M9 OMV vaccine provides significant protection against pneumonic melioidosis. Mice (*n* = 10 per group) were immunized with 10 μg M9 OMVs, 10^6^ cfu live Bp82, or saline (sham). Animals were challenged with 8 x LD_50_ aerosolized *B. pseudomallei* strain K96243. Both M9 OMV-immunized mice and mice immunized with the live-attenuated Bp82 vaccine were significantly protected against *B. pseudomallei* infection (****p < 0.0001 by log rank Mantel–Cox test compared to sham). There was no significant difference between M9 OMV and live Bp82 vaccinated mice (*p* = 0.14, hazard ratio 7.8, CI 0.48–125.1). Results shown are representative of two independent experiments.

The absence of toxicity and presence of potential immune protein targets indicated that M9 OMVs may constitute a safe and effective vaccine platform. To test this, we next compared the protective efficacy of the M9 OMV versus the live Bp82 strain (from which the OMVs are derived) in a comparator experimental immunization and challenge study. For these experiments, we tested the new M9 OMV formulation in C57Bl/6 mice since this strain has become the preferred rodent model for evaluation of *B. pseudomallei* vaccine candidates^5^. Mice were immunized subcutaneously twice, 3 weeks apart, with M9 OMVs or live Bp82 and challenged one month later with an aerosolized dose (8 x LD_50_) of virulent *B. pseudomallei* strain K96243 that would achieve complete mortality in control mice. There were no signs of reactogenicity at the injection site for any animal, and immunized animals showed no signs of discomfort or stress over the immunization period. These results are in agreement with our observations above that M9 OMVs are non-toxic. Notably, both the M9 OMVs and the live vaccine strain provided significant protection against an otherwise completely lethal infection (Fig. 3). There was no significant difference in the overall survival of M9 OMV- versus live Bp82-vaccinated animals. No bacteria were detected (limit of detection – 10 cfu) in the spleens harvested from survivors in the vaccinated groups at the 30 day study endpoint (not shown). These results indicate that the acellular M9 OMV vaccine can induce protective immunity similar to that of a live, replicating strain in a murine model of pneumonic melioidosis.

### Mice immunized with M9 OMVs induce both humoral and cellular immune responses

**Fig. 4:**
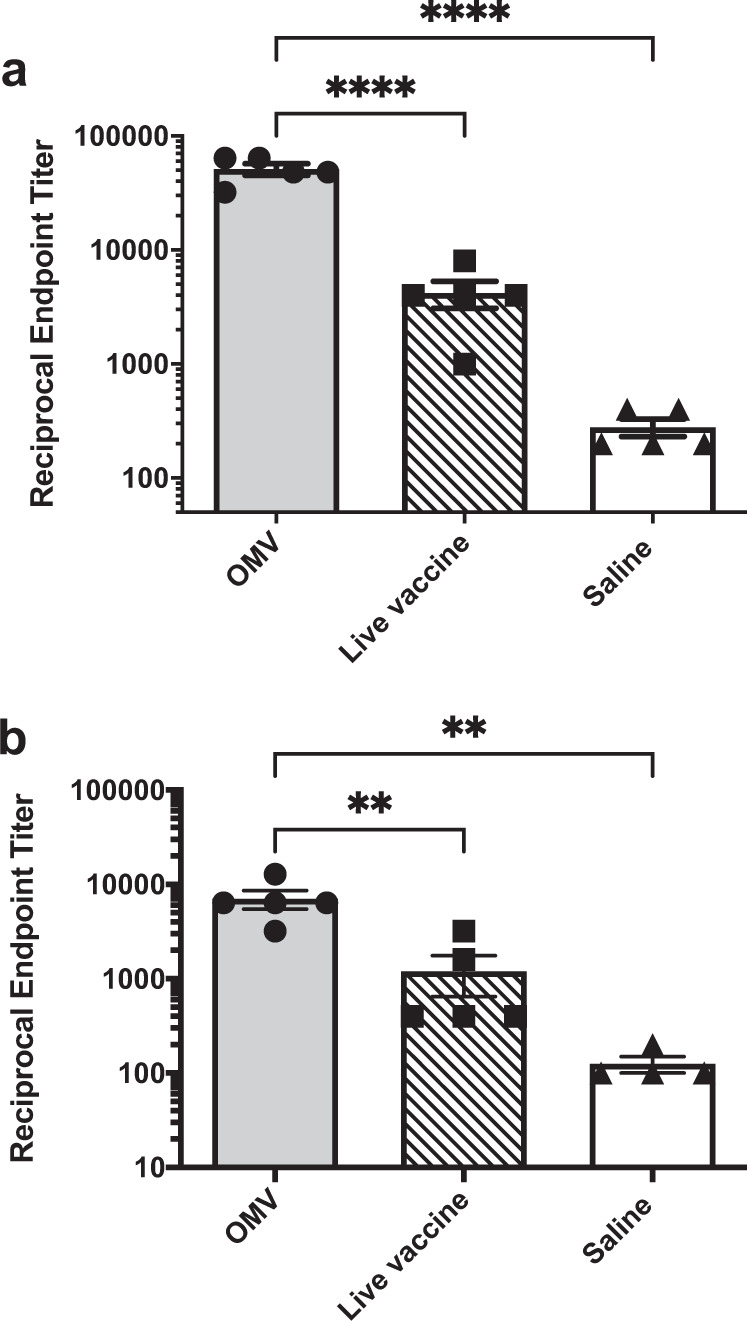
OMV immunization induces higher titers of IgG than live vaccine. Mean reciprocal endpoint titers of serum IgG were determined for mice (*n* = 5 per group) immunized with M9 OMVs, live-attenuated Bp82 vaccine, or sham by ELISA using microtiter plates coated with **a** M9 OMVs or **b** heat-inactivated M9-grown Bp82 bacteria (*p < 0.05, **p < 0.01, ***p < 0.001, ****p < 0.0001. Statistical analysis was performed using one-way ANOVA with Tukey’s multiple comparisons test. Results are representative of two independent experiments.

**Fig. 5:**
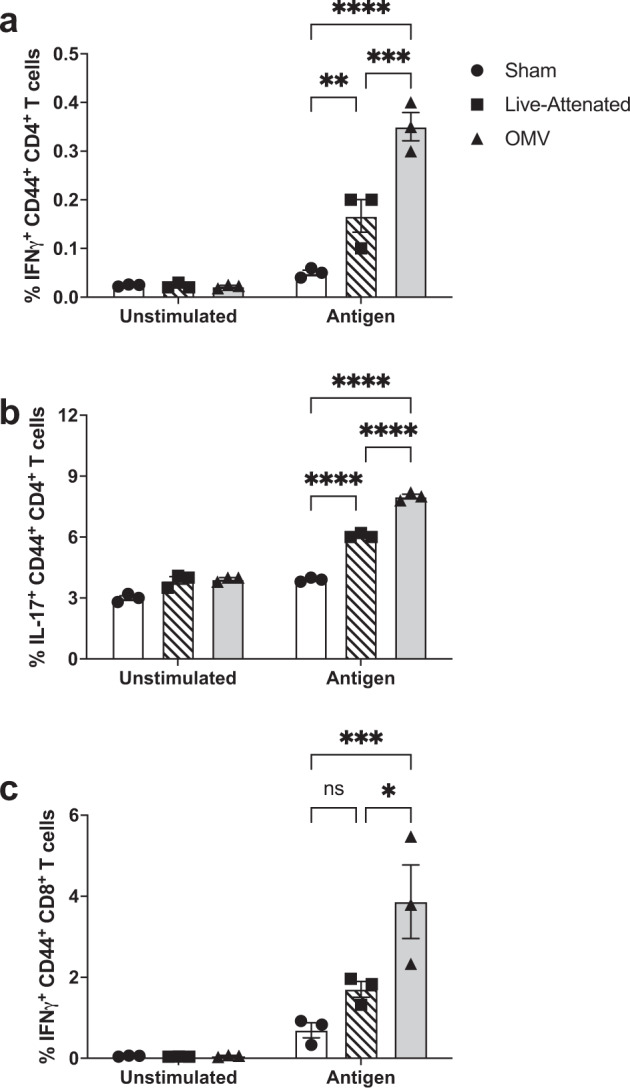
Immunization with M9 OMVs induces memory CD4 and CD8 T-cell responses. **a**–**b** Antigen-specific CD44 + CD4 + T cell and **c** CD8 + T-cell responses were measured in the spleens of mice (*n* = 3 per group) immunized with M9 OMV, live-attenuated vaccine, or sham (saline). Splenocytes were restimulated with a-CD28 and heat-inactivated Bp82 grown in M9 (antigen). PMA/ionomycin-treated and unstimulated groups were used as controls. Cytokine-producing T cells were assessed by intracellular cytokine staining and flow cytometry. *p < 0.05, **p < 0.01, ***p < 0.001, ****p < 0.0001. ns = not significant. Statistical analysis was performed using two-way ANOVA with Tukey’s multiple comparisons test. Results are representative of two independent experiments.

The compelling protective efficacy of the M9 OMV vaccine relative to immunization with a live, replicating vaccine prompted us to next examine the humoral and cellular immune responses elicited by each vaccine. As shown in Fig. 4, M9 OMV immunization induced significantly more serum IgG specific for both OMVs and whole inactivated bacteria compared to the live vaccine (Fig. 4a, b). Next, we compared the cellular immune responses elicited by M9 OMV and live vaccine using a T-cell cytokine response recall assay. Mice immunized with either live or M9 OMV vaccine demonstrated a significant increase in IFN-γ and IL-17 producing CD4^+^ T cells (Fig. 5a, b). However, M9 OMV vaccinated mice produced significantly higher numbers of cytokine-secreting CD4 T cells compared to the live vaccine. Lastly, mice immunized with M9 OMVs displayed an increase in IFN-γ producing CD8^+^ T cells, whereas mice immunized with the live vaccine did not (Fig. 5c). Taken together, these data indicate that the M9 OMV vaccine elicited both humoral and cellular immune responses that exceeded the response to the live parent strain in our experimental system.

### M9 OMVs activate antigen-presenting cells

**Fig. 6:**
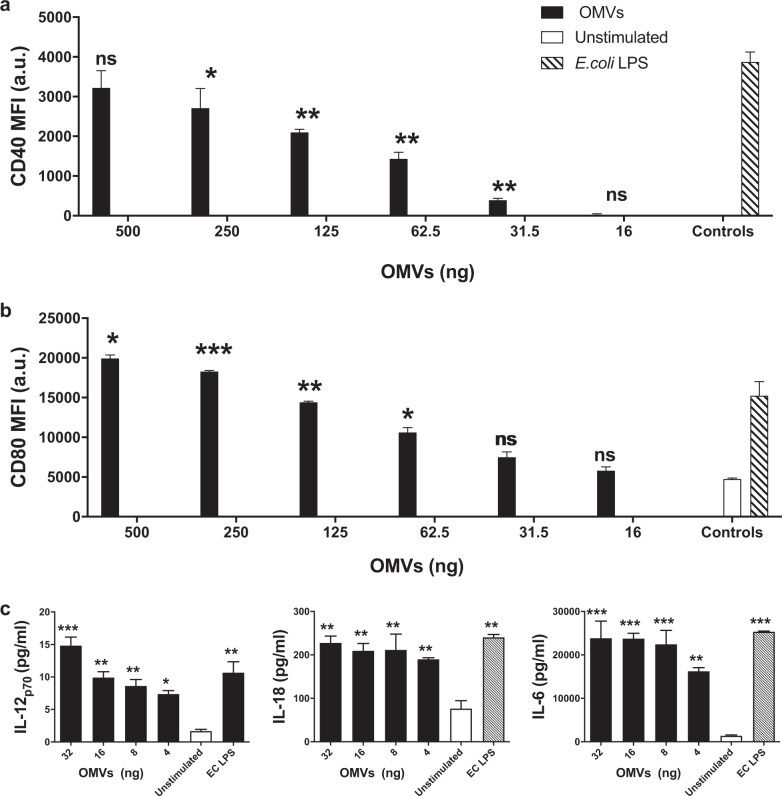
OMVs activate dendritic cells in vitro. BMDCs were treated with decreasing doses of OMVs or 5 ng/ml *E. coli* LPS as a positive control or left unstimulated as a negative control for 24 h. Median fluorescence intensity (MFI) was measured for **a** CD40 and **b** CD80 by flow cytometry. **c** Cytokines IL-12p70, IL-18, and IL-6 in the culture supernatants were measured by multiplex assay. ns = not significant, *p < 0.05, **p < 0.01, ***p < 0.001 compared to unstimulated by Tukey’s multiple comparison test. Results are representative of three independent experiments.

**Fig. 7:**
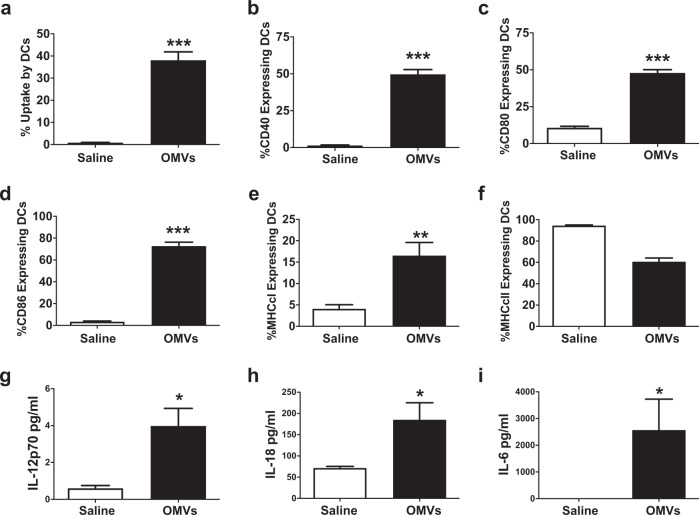
OMVs activate dendritic cells in vivo. Mice (*n* = 5 per group) were injected intraperitoneally with 10 μg CFSE-labeled OMVs or saline and cellular exudate was harvested 6 h later by peritoneal lavage. **a** The percentage of DCs harboring OMVs and expressing **b** CD40, **c** CD80, **d** CD86, **e** MCH class I, and **f** MHC class II was assessed by flow cytometry. The levels of cytokines **g** IL-12p70, **h** IL-18, and **i**) IL-6 present in lavage fluid were measured by multiplex assay. *p < 0.05, **p < 0.01, ***p < 0.001 compared to saline by Student’s *t* test. Results are representative of two independent experiments.

The remarkable ability of M9 OMVs to elicit both CD4^+^ and CD8^+^ T cells in vaccinated mice in the absence of exogenous adjuvant suggested that OMVs were activating antigen-presenting cells that could drive cellular immune responses. To test this, OMVs were added to murine bone marrow-derived dendritic cells (DC) and DC activation was assessed by upregulation of maturation and co-stimulatory markers and cytokine secretion. OMVs demonstrated a dose-dependent ability to drive DC maturation and activation as evidenced by increased expression of the maturation marker CD40 and co-stimulatory marker CD80 (Fig. 6a,b). Furthermore, OMVs stimulated DC production of Th1- and Th17-polarizing cytokines, including IL-12p70, IL-18, and IL-6, respectively (Fig. 6c). To confirm our in vitro observations, we next assessed the ability of OMVs to drive DC activation in vivo. As shown in Fig. 7a, greater than 30% of DCs harvested 6 h after intraperitoneal injection contained CFSE-labeled OMVs indicating they had taken up OMVs in the peritoneum. DCs from mice injected with OMVs displayed significant increases in CD40 (Fig. 7b), co-stimulatory markers CD80 and CD86 (Fig. 7c, d), and expression of MHC class I (Fig. 7e), but not MHC class II (Fig. 7f), compared to saline injected mice. Similar to our in vitro observations, IL-12p70, IL-18, and IL-6 cytokines were observed in the lavage fluid (Fig. 7g–i). These results indicate that OMVs possess inherent adjuvanticity through their ability to activate DCs.

## Discussion

Live attenuated vaccines have long been considered the gold standard for achieving protection against *B. pseudomallei* and other intracellular bacteria^5^. This is partly due to their ability to elicit both antibody and cellular immune responses against multiple target antigens, and in particular, to antigens expressed during different phases of infection. Here, we demonstrate that *B. pseudomallei* OMVs can be enriched with intracellular stage-specific antigens by mimicking the macrophage environment during bacterial culture. This M9 OMV vaccine provided complete protection in mice for up to 30 days and paralleled the protection elicited by the live parent vaccine strain. To our knowledge, this is the first demonstration of similar protection between an acellular, non-replicating vaccine and a live attenuated vaccine in a direct comparator lethal *B. pseudomallei* aerosol challenge model. It is possible that either the OMV or live Bp82 vaccine is more protective but we were unable to determine this with the restricted sample size and further study is required. Nonetheless the protection observed in M9 OMV vaccinated mice is very promising, particularly since 100% of OMV vaccinated mice survived and no bacteria were detected in the tissues of survivors.

Intuitively, it is logical that proteins expressed by bacteria upon human infection would be more appropriate targets than proteins expressed during growth in a liquid nutrient rich media like LB. Work by Champion et al. showed that enrichment of a multivalent subunit vaccine preparation with chronic stage-specific antigens could enhance vaccine efficacy against *B. pseudomallei*^26^. It is noteworthy that 95 of the 103 M9 OMV proteins in our studies were present in >95% of the *B. pseudomallei* isolates examined. Genes that are essential for pathogenicity should be mostly conserved among *B. pseudomallei* clinical isolates with some noted exceptions^27^. Within the set of genomes analyzed, ~50% of the genomes were collected from melioidosis patients suggesting that many of these genes may be required for host colonization and infection. The observation that M9 OMVs contain a large number of these highly conserved proteins, including proteins required for pathogenicity, may indicate that the M9 OMV vaccine is likely to confer broad coverage against diverse pathogenic *B. pseudomallei* strains. Furthermore, human melioidosis patients mount antibody responses to a number of M9 OMV proteins including, but not limited to, Hcp-1^28^, OmpA^29,30^, and FlgK^31^, suggesting they could each contribute to vaccine efficacy. Although we detected Hcp-1 in M9 OMVs by LC-MS and Western blot, antibody responses to rHcp-1 were undetectable by ELISA (not shown). The location and abundance of M9 OMV proteins is likely to influence their contribution to vaccine efficacy, and further studies are needed to elucidate the antigenic determinants of protection for the M9 OMV vaccine. Nonetheless, the antibody responses elicited by the OMV vaccine most likely account for the significant protection observed since we and others have shown that antibodies are sufficient for protection in mouse models of melioidosis^29,32–34^.

In addition to antibodies, the M9 OMV vaccine elicited a mixed Th1/Th17 CD4^+^ T cell response as well as a CD8^+^ T-cell memory response in C57BL/6 mice. The ability of the OMV vaccine to drive both helper and cytotoxic T cells is significant because although T cells appear dispensable in mouse models, it is likely that cellular immunity will be important for complete vaccine efficacy against melioidosis in humans^35,36^. Both live and non-replicating vaccine candidates for *B. pseudomallei* have been shown to induce Th1 immune responses, as evidenced by CD4 T-cell IFN-γ production, in mouse models^32,37^. It is well established that vaccine-elicited IFN-γ-producing CD4 T cells drive the antibacterial effector functions of macrophages to control or eliminate facultative intracellular bacteria. Both the live and OMV vaccines examined in this study promoted IFN-γ-producing CD4 T cells. These findings corroborate previous work that demonstrated IFN-γ-production by CD4 T cells after immunization with the live vaccine strain Bp82^32,33^. In addition, both live- and OMV-immunized mice produced CD4 T cells that secreted IL-17. The role of Th17 immunity in protection against human melioidosis is not well defined. *B. pseudomallei* infection can occur through multiple routes of infection, including inhalation and ingestion, and Th17 responses can be beneficial during mucosal bacterial infection^38^. In particular, neutrophil recruitment by IL-17 to sites of mucosal infection are often correlated with improved clearance of bacteria^39^.

A protective role for IFN-γ producing CD8 T cells in melioidosis is supported by several studies^36^, and the cytosolic niche of *B. pseudomallei* would suggest that CD8 T-cell recognition of bacterial peptides presented on MHC class I would be important for detecting infected host cells. Cytotoxic T-cell response elicited by the M9 OMV vaccine could thus help to control or eliminate any bacteria that avoid initial antibody clearance. The ability of M9 OMVs to elicit CD8 T cells is quite remarkable considering that most non-replicating vaccines are unable to do so^40^. We speculate that the successful ability of the OMV vaccine to elicit CD8 T-cell responses is due to the noted ability of OMVs to deliver antigenic cargo to the cytosolic compartment of host cells^41,42^, which would then facilitate OMV antigen presentation via MHC class I. Alternatively, it is possible that OMV antigens are recognized through cross-presentation by antigen-presenting cells. Finally, we do not yet know the antigen specificity of the T-cell responses. The M9 OMV vaccine contains several proteins, such as AhpC and BopE, which have been associated with protective cellular memory responses in melioidosis survivors^43^. Screening of T CD8 or CD4 T-cell epitopes for particular antigens was beyond the scope of the current work, and further study is needed to determine the extent of the antigen-specific immune responses, such as those to T3SS-3, T6SS-1, and other proteins present in the M9 OMV formulation.

One of the most compelling observations regarding the OMV vaccine is its ability to elicit all arms of the immune response without a requirement for any exogenous adjuvant. Here we show for the first time that OMVs are readily taken up by dendritic cells in vivo and drive DC activation, maturation, and cytokine secretion. OMVs increased MHC class I expression by DCs in vivo lending support to its ability to stimulate CD8 T cells after immunization. We were unable to detect a significant increase in MHC class II expression because the baseline expression was nearly 100% in control-treated animals. However, OMV treatment resulted in the production of Th1- and Th-17- promoting cytokines in vitro and in vivo, which can support the development of CD4 T cells like those we observed after immunization. While we did not directly compare DC responses to OMVs and live Bp82, it is well-established that live-attenuated vaccines drive DC activation leading to robust T-helper and cytotoxic T-cell immune responses^44,45^. Thus, OMV activation of dendritic cells in vivo may account for the CD4 and CD8 T-cell responses observed following OMV vaccination.

In conclusion, the second-generation M9 OMV vaccine appears to be an excellent candidate based on its lack of toxicity, potent adjuvanticity and immunogenicity, and protective efficacy similar to that of a live-attenuated vaccine. Furthermore, purification of OMVs from bacteria grown in a chemically defined minimal media will facilitate current Good Manufacturing Practice (cGMP) production and avoid any potential contamination with animal-based additives. Additionally, the M9 OMV vaccine may provide a promising alternative to live attenuated vaccines, particularly for use in immunocompromised populations.

## Methods

### Ethics statement

This study was performed in strict accordance with the Guide for the Care and Use of Laboratory Animals of the National Institutes of Health (NIH). The protocols were approved by the Tulane University Institutional Animal Care and Use Committee. The Tulane National Primate Research Center (TNPRC) is fully accredited by the Association for the Assessment and Accreditation of Laboratory Animal Care-International. Prior to challenge, mice were transferred to an Animal Biosafety Level (ABSL)-3 facility at the TNPRC and allowed to acclimate for one week prior to challenge. For survival studies, death was not used as an endpoint. Mice were observed at least three times daily, including weekends. Euthanasia was performed on terminally ill mice and at the study endpoints by CO_2_ overdose and confirmed by cervical dislocation.

### Bacterial strains and growth conditions

*B. pseudomallei* strain Bp82 was kindly provided by Herbert Schweizer and is a ∆*purM* derivative of *B. pseudomallei* strain 1026b^13^. *E. coli* strain M15 was obtained from Qiagen. Bacteria were cultured from glycerol stocks immediately prior to use and single colonies were selected from freshly streaked Luria Broth (LB) agar plates. For the challenge experiments, overnight cultures of *B. pseudomallei* K96243 (BEI resources) were diluted 1:100 in fresh LB with 4% glycerol (MilliporeSigma) and incubated with shaking at 37 °C until OD_600_ reached 0.75. Cultures were centrifuged and bacterial pellets were washed and re-suspended to achieve the desired concentration. For live vaccination with Bp82, overnight cultures were diluted 1:100 in fresh LB supplemented with 100 μg/mL adenine hydrochloride (MilliporeSigma, St. Louis, MO, USA) and 5 μg/mL thiamine hydrochloride (MilliporeSigma) and incubated with shaking at 37 °C until the OD_600_ reached 1.0.

For bacterial growth in minimal media, a single colony of Bp82 was inoculated into M9CG media consisting of M9 minimal salts agar (BD Difco) supplemented with 0.4% glucose (Sigma) and 0.5% casamino acids (Amresco)^20^, with 100 μg/ml adenine (Sigma) and 5 μg/ml thiamine hydrochloride (Sigma) (hereafter referred to as M9). Iron levels in M9 media were determined using the iron quantification reagent Ferrozine (ε_562nm_, 27.9 mM^−1^ cm^−1^) in the presence of excess cysteine, as performed previously^46^. The level of zinc in M9 media was quantified by the appearance of the Zn: 4-(2-pyridylazo)resorcinol complex peak at 490 nm on a BioTek Epoch 2 plate reader as previously described^46^. Iron and zinc levels in the M9 media were 0.42 and 0.01 uM, respectively.

For bacterial heat-inactivation, overnight cultures were diluted 1:100 in 50 ml of M9CG and grown for 6 h. The bacterial cultures were centrifuged at 9000 x *g* for 10 min, and the bacterial pellets were resuspended in 5 ml dH_2_O and heat-inactivated for 4 h by incubation at 60 °C. To confirm inactivation, 10% of the bacterial suspension was plated onto LB agar. A Bradford assay was used to determine protein concentration after inactivation, and heat-inactivated bacteria were stored at −20 °C until use in ELISA and splenocyte restimulation assay.

### OMV purification

OMVs were purified as previously described^8^ with minor modifications. *B. pseudomallei* strain Bp82 or *E. coli* were freshly streaked from a glycerol stock onto LB agar and incubated for 48–72 h at 37 ^◦^C. For preparation of M9 OMVs, an individual colony of Bp82 was inoculated into M9CG and incubated at 37 ^◦^C for 16–18 h. The overnight cultures were diluted 1:100 into M9CG media and incubated at 37 ^◦^C for 16–18 h until late log phase (OD_600_ 4.5–5.0). Intact bacteria were pelleted by centrifugation (6000 × *g* for 30 min at 4 ^◦^C) using an SLA-1500 fixed angle rotor. Following centrifugation, the supernatant was filtered through a 0.22 μm polyethersulfone (PES) membrane (MilliporeSigma) to remove any remaining bacteria or large bacterial fragments. Absence of bacterial contamination was verified by incubating 1 mL per liter of supernatant on LB agar for 48–72 h at 37 ^◦^C. OMVs were precipitated by incubating with 1.5 M ammonium sulfate (Fisher Scientific, Pittsburgh, PA, USA) overnight and then harvested by centrifugation (11,000 × *g*, 45 min, 4 ^◦^C) using an SLA-1500 rotor. Crude vesicles were resuspended in 60% sucrose (MilliporeSigma) in 30 mM Tris-HCL pH 8.0, layered at the bottom of 35–60% density gradient, and subjected to ultracentrifugation (200,000 × *g*, 3 h, 4 ^◦^C) using a 50.2Ti rotor. Fractions of equal volume were removed from the top then evaluated by SDS-PAGE to visualize protein profiles by Coomassie blue staining as previously described^7^. Fractions containing identical protein profiles were pooled and subjected to ultracentrifugation (200,000 × *g*, 19 h, 4 ^◦^C) to obtain highly purified vesicles similar to those obtained from LB cultures (Supplementary Fig. 1). Purified vesicles were re-suspended in LPS-free water, visually confirmed by transmission electron microscopy, and quantitated by Bradford assay as previously described^8^.

### Liquid chromatography/mass spectrometry (LC/MS)

In order to determine the protein composition of the M9 OMV preparations, LC/MS was performed on the preparation by Dr. Chau-Wen Chou (Department of Chemistry, University of Georgia). For analysis, 100 μg of OMV was run by SDS-PAGE until the band reached the stacking portion of the gel. Bands were excised from the gel and incubated for 20 min with 25 mM ammonium bicarbonate in 50% acetonitrile. Proteins were then digested with 1 μg/sample Trypsin in 25 mM ammonium bicarbonate for 16 h at 37 °C. Peptides were extracted by incubating the samples with 100 μl extraction buffer, comprised of 0.1% formic acid in 50% acetonitrile, for 20 min, briefly spun and supernatant collected. This was then followed by an additional incubation for 20 min with 100% acetonitrile. Samples were then dehydrated by Eppendorf Vacufuge and resuspended in 10 μl 0.1% formic acid with 2% acetonitrile. Samples were run on a ThermoScientific Orbitrap Elite mass spectrometer for high resolution and high mass accuracy analysis. This was coupled with a nano HPLC. Results were provided as raw data, which was searched against the Bp K96243 proteome through the Basic Local Alignment Search Tool (BLAST) search engine.

### Western blot

The presence of Hcp-1 in the OMVs was assayed by Western blot. Ten μg of Bp82 LB OMV (control) or M9 OMV preparations were separated on a 4–20% SDS-PAGE gel (BioRad) alongside a protein molecular weight ladder (BioRad). After separation, the proteins were transferred from the gel to a nitrocellulose membrane (iBLOT) using the iBLOT system per manufacturer instructions. The membrane was then blocked in 1.5% bovine serum albumin (BSA) in tris-buffered saline with 1% Tween-20 (TBS-T) for 1 h, then washed and incubated overnight at 4 °C with rat anti-Hcp-1 sera (kindly provided by Drs. Mary Burtnick and Paul Brett), diluted 1:1000. The following day, membranes were washed with TBS-T and incubated for 1 h with HRP-conjugated goat anti-rat IgG (Abcam) diluted 1:2000 in TBS-T. Membranes were washed and developed with the Opti-4CN kit (BioRad) according to manufacturer instructions and imaged using a GE AI^600^ RBG Imager.

#### OMV protein comparison to coding regions in sequenced *B. pseudomallei* isolates

To determine conservation of the OMV proteins identified by mass spectrometry, the amino acid sequence was compared to coding regions in 407 sequenced *B. pseudomallei* with the large-scale BLAST score ratio (LS-BSR) pipeline^47^ using the tblastn aligner^48^. A BLAST bit score incorporates amino acid sequence identity and alignment length and compares this sequence to a reference. The reference for the LS-BSR analysis was *B. pseudomallei* 1026b, which is the parent isolate of Bp82^13^ used to generate the OMVs. All of the publicly available *B. pseudomallei* genomes were downloaded on January 3^rd^ 2018. To ensure quality coding region analysis, these genomes were then screened for sequencing and assembly quality. Low-quality genomes were not used since this can decrease accurate coding region prediction. This set of *B. pseudomallei* isolates contain genomes from 201 isolates collected from patients and the remaining 206 isolates were collected from the environment, animals, or unknown.

#### Infection of *Galleria mellonella* larvae

*G. mellonella* larvae were purchased from Carolina Biological Supply (Burlington, NC). Larvae weighing between 0.2 and 0.3 g were selected for experiments. For each experiment a total of 10 larvae were used per OMV to be tested. The larvae were infected by micro-injection (Hamilton Ltd) into the right foremost proleg with 10 ug OMV in 10 µl volume. For control groups, 10 larvae were inoculated with PBS and a further 10 were left uninoculated. The larvae were incubated at 37 °C and survival was recorded for 5 days. Larvae were scored as dead when they ceased moving and displayed complete melanization^49^.

#### Treatment of murine macrophages

The murine macrophage cell line RAW 264.7 (ATCC TIB-71) was obtained from the American Type Culture Collection (ATCC, Rockville, MD). Cells were maintained in Dulbecco’s modified Eagle’s medium (DMEM; Invitrogen) supplemented with 10% (vol/vol) heat-inactivated fetal bovine serum (FBS; Invitrogen) and a standard mixture of antibiotics (100 U/ml penicillin, 100 μg/ml streptomycin, and 250 μg/ml amphotericin B) at 37 °C under an atmosphere of 5% CO_2_. For cytotoxicity assays, RAW 264.7 cells were resuspended in DMEM supplemented with FBS (DMEM-10) and transferred into the wells of 24-well tissue culture plates. Wells were treated with 10 μg of M9 OMVs, 10 μg E. coli-derived OMVs, or PBS and incubated overnight. Treated RAW 264.7 cell supernatants were assayed for lactate dehydrogenase (LDH) release by using a CytoTox 96 nonradioactive cytotoxicity assay kit (Promega). Maximum release was achieved by lysis of monolayers with Triton X-100 at a final concentration of 1% (vol/vol). The LDH released by uninfected cells was designated the spontaneous release. Cytotoxicity was calculated as follows: percent cytotoxicity = (test LDH release − spontaneous release)/(maximal release − spontaneous release).

### Mouse immunization and challenge experiments

Male and female C57BL/6 mice, 8 to 10 weeks old, were purchased from Charles River Laboratories (Wilmington, MA) and maintained 5 per cage in polystyrene microisolator units under pathogen-free conditions. Animals were fed rodent chow and water ad libitum and allowed to acclimate 1 week prior to use.

Mice were immunized subcutaneously with 10 μg of M9 OMVs diluted in 100 μl of sterile saline. Control animals were sham immunized with saline. For the live-attenuated vaccine, mice were immunized subcutaneously with 10^6^ colony-forming units (cfu) of live Bp82 bacteria. Mice were administered a booster dose three weeks after the initial dose. One month after the final boost, immunized and control mice (*n* = 10 per group) were challenged by small particle aerosol as previously described^50^ using starting concentrations in the range of of 1–5 × 10^8^ cfu/ml of *B. pseudomallei* K96243 (BEI Resources). Actual mean infectious doses delivered to the mice were determined by plating the inoculum and all glass impinger (AGI) aerosol sampler collections for each discrete run of the aerosol system. For the challenge experiment, mice received approximately 1500 cfu per mouse, equating to 8x multiplicity of the established LD_50_, respectively. Survival was monitored up to 30 days post infection. Spleens were harvested from mice that survived to the study endpoint in order to assess persistent bacterial infection. Tissues were aseptically removed from euthanized animals, individually placed in 1 ml 0.9% NaCl, and homogenized with sterile, disposable tissue grinders (Fisher Scientific). Ten-fold serial dilutions of spleen homogenates were plated on LB agar. Colonies were counted after incubation for 3 days at 37 °C and reported as cfu per spleen, with a lower limit of detection of 10 cfu.

### Assessment of antibody responses to vaccination

Four weeks after the final immunization, mice were sacrificed by CO_2_ asphyxiation. Blood was collected by cardiac puncture, centrifuged in serum separator tube (BD) at 7000 x *g* for 10 min, and sera was collected and stored at −80 °C until analysis by enzyme linked immunosorbent assay (ELISA). For ELISA assays, flat bottom 96-well polystyrene plates (Costar) were coated overnight at 4 °C with heat-inactivated Bp82 in coating buffer comprised of 0.1 M sodium bicarbonate + 0.2 M sodium carbonate + 0.1 g sodium azide in 500 mL sterile dH_2_O, at a concentration of 0.5 μg/well. Plates were then washed three times with PBS + 0.5% Tween 20, hereafter referred to as PBS-T. Samples were diluted serially in a dilution buffer of PBS-T. Serially diluted serum samples were added to the coated plates, and samples were incubated for 1 h at room temperature. Plates were washed 3 times with PBS-T. Detection by IgG ELISAs was performed using AKP-conjugated rabbit anti- mouse IgG (Sigma) as a secondary antibody, diluted 1:300 in dilution buffer and added at a volume of 100 μl/well, then incubated for 1 h at room temperature. Plates were then washed 5 times with PBS-T. For detection of IgG, p-nitro-phenyl-phosphate (Sigma) was dissolved in diethanolamine buffer at a concentration of 1 mg/ml and 100 μl of this solution was added to the wells. After development of the assay, the reaction was stopped using 50 μl/well 2 M NaOH. Plates were read immediately at 405 nm to determine optical density (OD). Results are expressed as the mean reciprocal endpoint titers. Endpoint titer was defined as the greatest dilution yielding an optical density (OD_405_) greater than three standard deviations above the mean OD_405_ for “blank” background wells.

#### Assessment of T-cell responses to vaccination

T-cell responses to immunization were assessed in spleens collected 2 weeks after the final immunization. Single cell suspensions were prepared by homogenizing organs on a 70 μm nylon cell strainer (Fisher) with a rubber syringe plunger from a 5 mL syringe (Fisher). The cell suspension was centrifuged at 460 × *g* for 10 min at 4 °C. Supernatant was decanted and the cells were resuspended in 2 mL ACK red blood cell lysis buffer (Invitrogen, Waltham, MA, USA) and incubated at room temperature for 3 min and the reaction stopped with 20 mL of RMPI (Gibco, Waltham, MA, USA) containing 10% fetal bovine serum (FBS, Atlanta Biologicals, GA, USA), hereafter referred to as 10% RPMI. Cells were then centrifuged at 300 × *g* for 10 min, supernatant was decanted, and the cells were resuspended in 5 mL 10% RPMI. The viable cells were counted on a Cellometer (Nexcelom Bioscience) using Trypan Blue (Sigma) and corrected to a final volume of 1 × 10^7^ cells/mL from which 1 × 10^6^ cells were added per well to a 96-well round bottom plate. Cells were stimulated with 0.4 μg purified anti-CD28 (eBioscience, Waltham, MA, USA) alone or together with 2.5 mg of OMVs or with 10 ng PMA (Sigma-Aldrich) and 100 ng Ionomycin (Sigma-Aldrich) for 2 h at 37 °C with 5% CO_2_. GolgiPlug (BD Biosciences, San Jose, CA, USA) was added to cells to immobilize intracellular cytokines and the incubation was allowed to proceed for a further 6 h. Cells were washed once by centrifugation at 400 × *g* for 3 min with 1× PBS. Cells were stained for viability using 0.1 μL Fixable Viability Dye-eFluor780 (eBioscience) for 30 min on ice then washed with sorter buffer (2% FCS, 1 mM EDTA, 0.1% Sodium Azide in 1× PBS) and incubated with 0.1 mL anti-CD16/32 FcBlock (eBioscience) in sorter buffer for 10 min on ice. Cells were labeled for 30 min on ice with fluorescently labeled antibodies (0.25 μg per sample) for analysis by flow cytometry as follows: CD3-BV605 (BD Biosciences; clone 1702), CD4-BV510 (BD Biosciences; clone M4-5), CD8-PE-Cy7 (eBioscience; clone .3–6.7), CD44-eF450 (eBioscience; clone M7), and a T-cell lineage negative redFluor710-labeled antibody cocktail consisting of the following markers: B220 clone A3-6B2, CD11b clone 1/70, CD11c clone 418, CD19 clone D3, F4/80 clone BM8.1 (Tonbo Biosciences, San Diego, CA, USA). Cells were washed twice with sorter buffer and incubated with Fixation/Permeabilization Buffer (BD Biosciences) for 60 min on ice. Cells were washed twice with Wash/Perm Buffer (BD Biosciences) and stained for intracellular cytokines using the antibodies anti-IL-17A-PerCP-Cy5.5 (eBioscience; clone eBio17B7) and anti-IFN-γ-PE (eBioscience; clone XMG1.2) overnight at 4 °C. Cells were washed twice in Wash/Perm and once in sorter buffer. Samples were acquired on an LSR Fortessa (BD Biosciences) and flow cytometry data was analyzed using FlowJo (Treestar, Ashland, OR, USA).

#### Activation of bone-marrow derived dendritic cells (BMDC)

For in vitro experiments, BMDCs were generated as described previously^51^. Cells were plated on day 10 at concentrations of 1 × 10^6^ cells/ml and stimulated 2 h later with decreasing doses of M9 OMVs, 5 ng/ml *E. coli* LPS (Sigma) or left unstimulated for 24 h. For the analysis of surface marker expression, cells were incubated with AQUA LIVE/DEAD fluorescent dye (0.5 μl) (Invitrogen) for 30 min. Cells were subsequently stained with anti-CD11c (PE-Cy7, eBioscience), anti-CD40 (APC, eBioscience), and anti-CD80 (PerCP-Cy5.5, BD Biosciences) then measured by flow cytometry as described above. The levels of IL-12p70, IL-18, and IL-6 cytokines in culture supernatants were measured by multiplex assay (BioRad).

For in vivo studies, C57Bl/6 mice were injected intraperitoneally with 100 μl saline control or 10 μg OMVs that were pre-labeled with CFSE (CellTrace, Life Technologies) according to the manufacturer’s instructions. After 6 h, cellular exudates were obtained by peritoneal lavage. Cells were stained with anti-CD11c, anti-CD40 (PE), anti-CD80 (PE-CF594), anti-CD86 (BV605), anti-MHC class I (APC), anti-MHC class II (PerCP Cy5.5), and analyzed by flow cytometry. The levels of IL-12p70, IL-18, and IL-6 cytokines in lavage fluid were measured by multiplex assay (BioRad).

#### Statistical analyses

Statistical analyses were performed using GraphPad Prism version 5.0 (GraphPad Software, San Diego, CA, USA). *p*-values < 0.05 were considered statistically significant.

### Reporting summary

Further information on research design is available in the Nature Research Reporting Summary linked to this article.

## Supplementary information

Supplementary Data

Reporting Summary

## Data Availability

All relevant accession codes are provided in Supplementary Table 1.

## Supplementary information

**Supplementary information** The online version contains supplementary material available at 10.1038/s41541-021-00281-z.

## Code availability

This is not applicable to this work.
