# Supplementary material for: Burkholderia pseudomallei OMVs derived from infection mimicking conditions elicit similar protection to a live-attenuated vaccine
Source: NPJ Vaccines. 2021 Jan 29;6:18. doi: 10.1038/s41541-021-00281-z (PMC7846723; doi:10.1038/s41541-021-00281-z)

Supplementary Table 1. Proteins identified in LB OMVs and M9 OMVs by LC-MS.

	protein and coding region information			LCMS presence		tBlastn LSBSR results			
**#**	**gi**	**Accession number**	**Protein**	**LB**	**M9CG**	**# of genomes at >=0.95 BSR**	**% >=0.95**	**# of genomes at >= 0.9 BSR**	**% >=0.9 BSR**
1	53716140	YP_106305.1	succinate dehydrogenase iron-sulfur subunit			407	100.0%	407	100.0%
2	53716718	YP_105075.1	lipoprotein			407	100.0%	407	100.0%
3	53717914	YP_106900.1	flagellar hook protein FlgE			407	100.0%	407	100.0%
4	53719150	YP_108136.1	hypothetical protein BPSL1516			407	100.0%	407	100.0%
5	53720358	YP_109344.1	oxidoreductase			407	100.0%	407	100.0%
6	53720376	YP_109362.1	hypothetical protein BPSL2766			407	100.0%	407	100.0%
7	53720900	YP_109886.1	S-adenosyl-L-homocysteine hydrolase			407	100.0%	407	100.0%
8	53723542	YP_103005.1	4-hydroxy-3-methylbut-2-en-1-yl diphosphate synthase			407	100.0%	407	100.0%

9	490656359	WP_004521349.1	acetyl-CoA acetyltransferase	[redacted]	407	100.0%	407	100.0%
10	126452465	YP_001066743.1	OMP85 family outer membrane protein	[redacted]	407	100.0%	407	100.0%
11	386860661	YP_006273610.1	OmpA family outer membrane protein	[redacted]	407	100.0%	407	100.0%
12	490689719	WP_004554082.1	flagellar hook-associated protein FlgK	[redacted]	407	100.0%	407	100.0%
13	497624314	WP_009938498.1	membrane protein	[redacted]	407	100.0%	407	100.0%
14	685798421	AIP79574.1	mucB/RseB family protein	[redacted]	407	100.0%	407	100.0%
15	686980740	KGD57528.1	flagellar biosynthesis anti-sigma factor FlgM	[redacted]	407	100.0%	407	100.0%
16	705765759	KGS98746.1	ahpC/TSA family protein	[redacted]	407	100.0%	407	100.0%
17	740987408	WP_038770950.1	hypothetical protein	[redacted]	407	100.0%	407	100.0%
18	760210213	KIX56657.1	protein tolQ	[redacted]	407	100.0%	407	100.0%
19	760217559	KIX63860.1	general secretion pathway protein GspG	[redacted]	407	100.0%	407	100.0%

20	760219919	KIX66127.1	preprotein translocase subunit SecA	[redacted]	[redacted]	407	100.0%	407	100.0%
21	740967818	WP_038752284.1	flagellin	[redacted]	[redacted]	407	100.0%	407	100.0%
22	490297290	WP_004192742.1	outer membrane protein W	[redacted]	[redacted]	406	99.8%	407	100.0%
23	740962478	WP_038747038.1	TonB-dependent receptor	[redacted]	[redacted]	406	99.8%	407	100.0%
24	53723306	YP_112291.1	heat shock protein 20	[redacted]	[redacted]	406	99.8%	407	100.0%
25	53719705	YP_108691.1	osmolarity response regulator		[redacted]	407	100.0%	407	100.0%
26	53720482	YP_109468.1	hypothetical protein BPSL2874		[redacted]	407	100.0%	407	100.0%
27	126454430	YP_001066470.1	competence lipoprotein ComL		[redacted]	407	100.0%	407	100.0%
28	490689210	WP_004553602.1	N-acetylmuramoyl-L-alanine amidase domain-containing protein		[redacted]	407	100.0%	407	100.0%
29	685788854	AIP70010.1	cheB methylesterase family protein		[redacted]	407	100.0%	407	100.0%
30	705767187	KGT00175.1	BON domain protein		[redacted]	407	100.0%	407	100.0%

31	740930838	WP_038715872.1	hypothetical protein, partial			407	100.0%	407	100.0%
32	740943455	WP_038728461.1	LuxR family transcriptional regulator			407	100.0%	407	100.0%
33	740960650	WP_038745222.1	oxidoreductase			407	100.0%	407	100.0%
34	760210258	KIX56702.1	mechanosensitive ion channel protein MscS			407	100.0%	407	100.0%
35	760217996	KIX64280.1	H-type lectin domain protein			407	100.0%	407	100.0%
36	53716717.00	YP_105076.1	hypothetical protein BMAA0256			407	100.0%	407	100.0%
37	490301346	WP_004196744.1	membrane protein			407	100.0%	407	100.0%
38	685791441	AIP72597.1	flagellar P-ring family protein			407	100.0%	407	100.0%
39	740961148	WP_038745713.1	thioredoxin reductase			407	100.0%	407	100.0%
40	386863130	YP_006276079.1	penicillin-binding protein 6			406	99.8%	406	99.8%
41	53716763	YP_105780.1	porin OpcP1			406	99.8%	406	99.8%

42	53719081	YP_108067.1	lipoprotein	[redacted]	406	99.8%	406	99.8%
43	53720130	YP_109116.1	hypothetical protein BPSL2520	[redacted]	406	99.8%	406	99.8%
44	53720946	YP_109932.1	lipoprotein	[redacted]	406	99.8%	406	99.8%
45	124381615	YP_001025599.1	lipoprotein NlpD	[redacted]	406	99.8%	406	99.8%
46	126440935	YP_001060144.1	lipoprotein	[redacted]	406	99.8%	406	99.8%
47	490693230	WP_004557221.1	translocation protein TolB	[redacted]	406	99.8%	406	99.8%
48	760221512	KIX67671.1	alkyl hydroperoxide reductase	[redacted]	406	99.8%	406	99.8%
49	740936864	WP_038721884.1	BON domain protein, partial	[redacted]	406	99.8%	406	99.8%
50	53719908	YP_108894.1	phasin-like protein	[redacted]	405	99.5%	406	99.8%
51	53720930	YP_109916.1	flagellar hook-associated protein	[redacted]	405	99.5%	406	99.8%
52	490689459	WP_004553834.1	phenylacetic acid degradation protein paaN	[redacted]	406	99.8%	406	99.8%

53	740970097	WP_038754535.1	stress responsive protein			406	99.8%	406	99.8%
54	760221062	KIX67237.1	iron transporter			406	99.8%	406	99.8%
55	760221402	KIX67561.1	thioredoxin			406	99.8%	406	99.8%
56	740965554	WP_038750079.1	BapC protein			406	99.8%	406	99.8%
57	740952941	WP_038737603.1	flagellar basal body rod protein FlgG			406	99.8%	406	99.8%
58	740942950	WP_038727959.1	exopolysaccharide biosynthesis protein [Burkholderia pseudomallei]			404	99.3%	406	99.8%
59	685713146	AIP00200.1	bacterial regulatory helix-turn-helix, lysR family protein			406	99.8%	406	99.8%
60	685734648	AIP15947.1	universal stress family protein A			405	99.5%	406	99.8%
61	13649898	AAK37506.1	beta-lactamase precursor			405	99.5%	405	99.5%
62	53718085	YP_107071.1	preprotein translocase subunit SecB			405	99.5%	405	99.5%

63	53720009	YP_108995.1	non-hemolytic phospholipase C	[redacted]	405	99.5%	405	99.5%
64	53720132	YP_109118.1	outer membrane protein A	[redacted]	405	99.5%	405	99.5%
65	53720307	YP_109293.1	chaperonin GroEL	[redacted]	405	99.5%	405	99.5%
66	490688917	WP_004553327.1	outer membrane porin	[redacted]	405	99.5%	405	99.5%
67	490688936	WP_004553344.1	outer membrane porin	[redacted]	405	99.5%	405	99.5%
68	497612727	WP_009926911.1	x-prolyl-dipeptidyl aminopeptidase	[redacted]	405	99.5%	405	99.5%
69	740934573	WP_038719593.1	heat shock protein 90, partial	[redacted]	405	99.5%	405	99.5%
70	741010714	WP_038793102.1	x-prolyl-dipeptidyl aminopeptidase	[redacted]	405	99.5%	405	99.5%
71	760220443	KIX66639.1	molecular chaperone GroES	[redacted]	405	99.5%	405	99.5%
72	685723194	AIP10245.1	transglycosylase SLT domain protein	[redacted]	405	99.5%	405	99.5%
73	490672272	WP_004537261.1	universal stress family protein	[redacted]	405	99.5%	405	99.5%

74	53716314	YP_106342.1	chitin binding domain-containing protein	[redacted]	404	99.3%	404	99.3%
75	53719357	YP_108343.1	arginine deiminase	[redacted]	404	99.3%	404	99.3%
76	760200234	KIX46960.1	peptidase	[redacted]	404	99.3%	404	99.3%
77	121598480	YP_993774.1	serine protease, MucD	[redacted]	402	98.8%	404	99.3%
78	490656961	WP_004521951.1	hypothetical protein BURPS668_3636	[redacted]	404	99.3%	404	99.3%
79	53718291	YP_107277.1	acyl-CoA dehydrogenase oxidoreductase	[redacted]	404	99.3%	404	99.3%
80	685732767	AIP14067.1	Rhs element Vgr family protein	[redacted]	404	99.3%	404	99.3%
81	740961815	WP_038746378.1	Hcp	[redacted]	404	99.3%	404	99.3%
82	740986062	WP_038769665.1	cyclophilin, partial	[redacted]	404	99.3%	404	99.3%
83	740989003	WP_038772422.1	type VI secretion protein	[redacted]	404	99.3%	404	99.3%
84	740958473	WP_038743097.1	fimbrial protein [Burkholderia pseudomallei]	[redacted]	402	98.8%	403	99.0%

85	685722491	AIP09542.1	hypothetical protein DP55_1535			403	99.0%	403	99.0%
86	740961813	WP_038746376.1	EvpB family type VI secretion protein			403	99.0%	403	99.0%
87	53720597	YP_109583.1	lipoprotein			402	98.8%	402	98.8%
88	497617556	WP_009931740.1	molecular chaperone DnaK, partial			402	98.8%	402	98.8%
89	386863036	YP_006275985.1	chitinase			400	98.3%	401	98.5%
90	740954837	WP_038739472.1	TonB-dependent receptor			401	98.5%	401	98.5%
91	685713863	AIP00917.1	phoPQ-activated pathogenicity-related family protein			401	98.5%	401	98.5%
92	685721129	AIP08181.1	guanine nucleotide exchange factor BopE			401	98.5%	401	98.5%
93	741005500	WP_038787980.1	beta-N-acetylhexosaminidase			400	98.3%	401	98.5%
94	740924102	WP_038709237.1	lytic transglycosylase			399	98.0%	400	98.3%
95	490663145	WP_004528135.1	flagellar hook-associated protein 2			400	98.3%	400	98.3%

96	126675025	ABO26350.1	BipB	[redacted]	400	98.3%	400	98.3%
97	490296495	WP_004191956.1	ecotin	[redacted]	400	98.3%	400	98.3%
98	685791047	AIP72203.1	alkylhydroperoxidase AhpD family core domain protein	[redacted]	400	98.3%	400	98.3%
99	685726459	AIO13703.1	Rhs element Vgr family protein	[redacted]	382	93.9%	398	97.8%
100	740933032	WP_038718052.1	membrane protein	[redacted]	394	96.8%	395	97.1%
101	740965308	WP_038749835.1	elongation factor Tu, partial	[redacted]	390	95.8%	391	96.1%
102	740987967	WP_038771479.1	metallo-beta-lactamase	[redacted]	385	94.6%	385	94.6%
103	76818186	YP_335355.1	bacteriolytic lipoprotein entericidin B-like protein	[redacted]	385	94.6%	385	94.6%
104	740985393	WP_038769023.1	flagellar L-ring protein FlgH	[redacted]	365	89.7%	384	94.3%
105	740931337	WP_038716360.1	universal stress protein UspA, partial	[redacted]	384	94.3%	384	94.3%
106	740936897	WP_038721917.1	elongation factor Tu, partial	[redacted]	377	92.6%	380	93.4%

107	740933277	WP_038718297.1	Type VI secreted protein			365	89.7%	370	90.9%
108	752526605	WP_041198169.1	membrane protein			239	58.7%	253	62.2%
109	685732488	AIP13788.1	putative gp27			63	15.5%	66	16.2%

Supplementary Figure 1. Visualization of purified Bp82 OMVs by transmission electron microscopy. Purified LB OMVs and M9 OMVs were negatively stained with 1% uranyl acetate and imaged by Transmission Electron Microscopy. OMVs contain a double membrane structures with an electron dense center and range between 25-200nm in diameter.

LB OMVs M9 OMVs

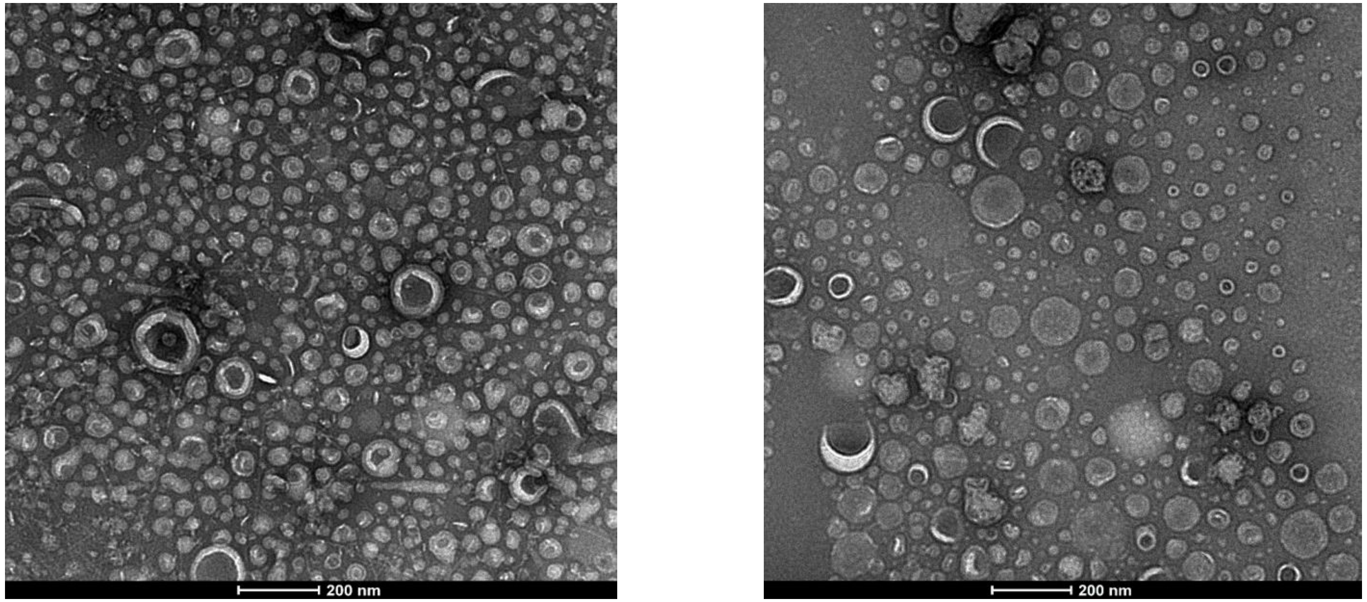

Supplementary Figure 2. Gating strategy for T cells. Single cell suspensions were acquired according to the methods and stained with fluorescently labeled antibodies for analysis by flow cytometry. Representative flow plots are shown for each population described in the manuscript. Cells were initially gated on single cells to eliminate doublets. Singlets were then gated on lymphocytes based on forward and side scatter properties followed by exclusion of dead cells. Next, T cell lineage negative cells were excluded including B cells, macrophages, and dendritic cells. Antigen experienced T cells were then identified using CD44 (Ag experience) and CD3 (pan T cell marker). CD4 and CD8 T cell populations within the total T cell pool were identified. For CD4 T cells, intracellular cytokine staining identified IFN-γ+ and IL-17+ T cells. For CD8 T cells, intracellular staining identified IFN-γ+ and granzyme B+ T cells. Cytokine gates were established based on gating on CD44 negative naïve T cells which do not express cytokines. All fluorochromes used are identified on the flow plots in combination with their respective markers and the numbers next to each gate represent their relative abundance as a percent of the total in that plot. All gates were set by staining each population with all markers minus the one being gated (fluorescence minus one or FMO).

Supplementary Figure 2. Gating strategy for T cells

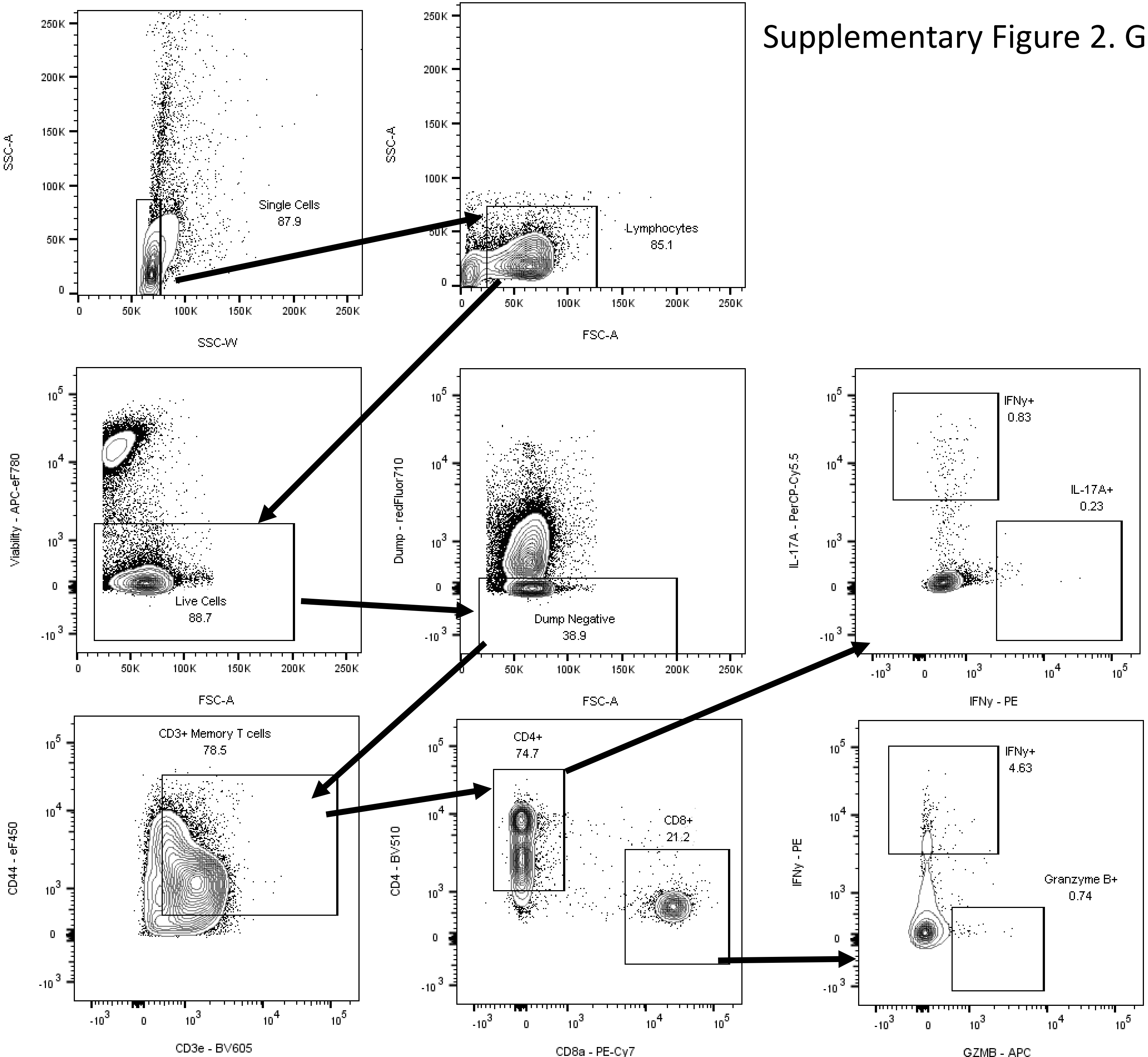

Supplementary Figure 3. Gating strategy for ex vivo dendritic cells. Single cell suspensions were acquired as above. Representative flow plots are shown for each population described in the manuscript. Cells were initially gated on all potential antigen presenting cells based on forward and side scatter properties followed by singlets and then exclusion of dead cells. Next, DC lineage negative cells were excluded including B cells, T cells, and NK cells. DCs were identified using the DC marker CD11c followed by exclusion of contaminating macrophages by gating on F4/80 negative CD11b negative cells. Finally, DC populations were phenotyped using CFSE labeled OMVs for uptake, CD40, MHC class I and II, CD80 and CD86. All fluorochromes used are identified on the flow plots in combination with their respective markers and the numbers next to each gate represent their relative abundance as a percent of the total in that plot. All gates were set by staining each population with all markers minus the one being gated (fluorescence minus one or FMO).

Supplementary Figure 3
Ex vivo DC Gating

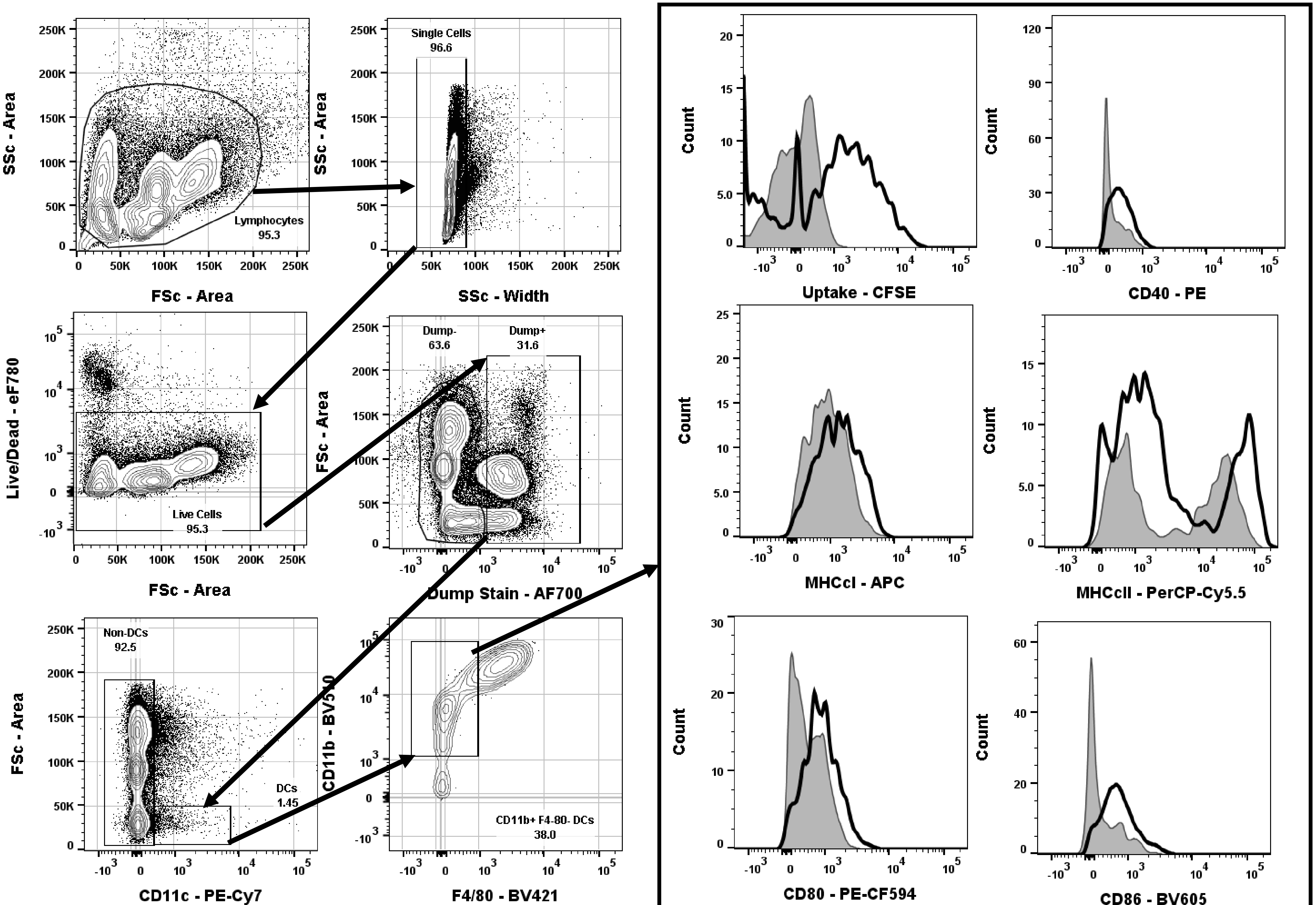

Supplementary Figure 4. Gating strategy for in vitro derived bone marrow dendritic cells (BMDCs). In vitro derived BMDCs were grown according to the methods. Representative flow plots are shown for each population described in the manuscript. Cells were initially gated on all potential antigen presenting cells based on forward and side scatter properties followed by singlets and then exclusion of dead cells. Next, DCs were identified using the DC marker CD11c and these cells were phenotyped using CD40 and CD80. All fluorochromes used are identified on the flow plots in combination with their respective markers and the numbers next to each gate represent their relative abundance as a percent of the total in that plot. All gates were set by staining each population with all markers minus the one being gated (fluorescence minus one or FMO).

Supplementary Figure 4. In vitro BMDCs Gating

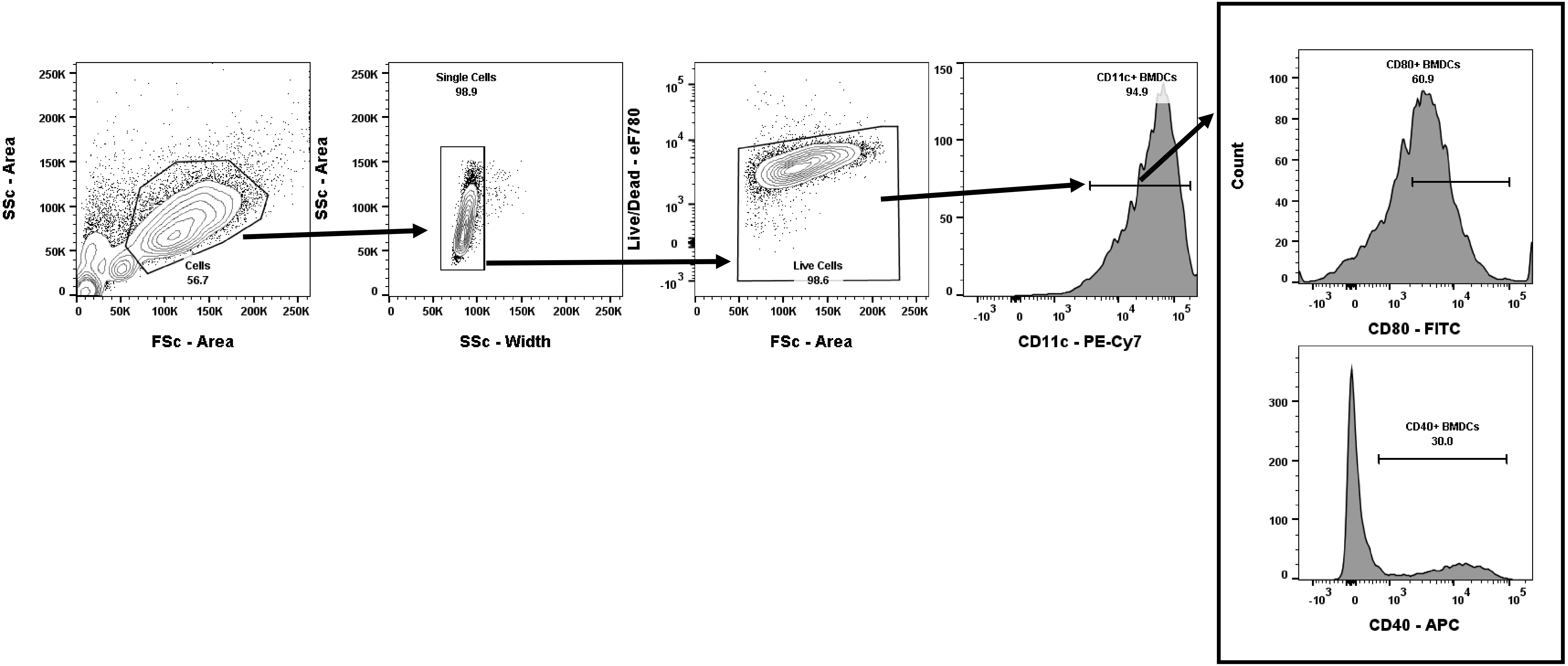

Supplement: Supplementary file 1 — Supplementary Data [file 41541_2021_281_MOESM1_ESM.pdf]
