# Supplementary material for: Burkholderia pseudomallei OMVs derived from infection mimicking conditions elicit similar protection to a live-attenuated vaccine
Source: NPJ Vaccines. 2021 Jan 29;6:18. doi: 10.1038/s41541-021-00281-z (PMC7846723; doi:10.1038/s41541-021-00281-z)
Supplement: Supplementary file 2 — Reporting Summary [file 41541_2021_281_MOESM2_ESM.pdf]

nature research

Corresponding author(s): Lisa A. Morici

Last updated by author(s): Nov 18, 2020

Reporting Summary

Nature Research wishes to improve the reproducibility of the work that we publish. This form provides structure for consistency and transparency in reporting. For further information on Nature Research policies, see our Editorial Policies and the Editorial Policy Checklist.

Statistics

For all statistical analyses, confirm that the following items are present in the figure legend, table legend, main text, or Methods section.

n/a	Confirmed	
☐	☒	The exact sample size (*n*) for each experimental group/condition, given as a discrete number and unit of measurement
☐	☒	A statement on whether measurements were taken from distinct samples or whether the same sample was measured repeatedly
☐	☒	The statistical test(s) used AND whether they are one- or two-sided<br>*Only common tests should be described solely by name; describe more complex techniques in the Methods section.*
☒	☐	A description of all covariates tested
☒	☐	A description of any assumptions or corrections, such as tests of normality and adjustment for multiple comparisons
☐	☒	A full description of the statistical parameters including central tendency (e.g. means) or other basic estimates (e.g. regression coefficient) AND variation (e.g. standard deviation) or associated estimates of uncertainty (e.g. confidence intervals)
☐	☒	For null hypothesis testing, the test statistic (e.g. *F*, *t*, *r*) with confidence intervals, effect sizes, degrees of freedom and *P* value noted<br>*Give P values as exact values whenever suitable.*
☒	☐	For Bayesian analysis, information on the choice of priors and Markov chain Monte Carlo settings
☒	☐	For hierarchical and complex designs, identification of the appropriate level for tests and full reporting of outcomes
☒	☐	Estimates of effect sizes (e.g. Cohen's *d*, Pearson's *r*), indicating how they were calculated

Our web collection on statistics for biologists contains articles on many of the points above.

Software and code

Policy information about availability of computer code

Data collection	No software used for data collection.
Data analysis	To determine conservation of the OMV proteins identified by mass spectrometry, the amino acid sequence was compared to coding regions in 407 sequenced B. pseudomallei with the large-scale BLAST score ratio (LS-BSR) pipeline [22] using the tblastn aligner [23]. Statistical analyses of data sets were performed using GraphPad Prism version 5.0 (GraphPad Software, San Diego, CA, USA)

For manuscripts utilizing custom algorithms or software that are central to the research but not yet described in published literature, software must be made available to editors and reviewers. We strongly encourage code deposition in a community repository (e.g. GitHub). See the Nature Research guidelines for submitting code & software for further information.

Data

Policy information about availability of data

All manuscripts must include a data availability statement. This statement should provide the following information, where applicable:

- Accession codes, unique identifiers, or web links for publicly available datasets
- A list of figures that have associated raw data
- A description of any restrictions on data availability

Accession codes for proteins are provided in a table in the supplementary data. There are no data restrictions.

Field-specific reporting

Please select the one below that is the best fit for your research. If you are not sure, read the appropriate sections before making your selection.

☒ Life sciences ☐ Behavioural & social sciences ☐ Ecological, evolutionary & environmental sciences

For a reference copy of the document with all sections, see nature.com/documents/nr-reporting-summary-flat.pdf

Life sciences study design

All studies must disclose on these points even when the disclosure is negative.

Sample size	The primary endpoint for sample size is based on a 2-fold difference of a given inflammatory mediator or cell type between a control group vs. an experimental group. Using this, a power of 90% (β = 0.1), two-sided analysis and a type I error rate of 5% (α = 0.05), an A priori sample size calculation for the Student's t-test estimates that each group would require a minimum of 4-6 mice per group.
Data exclusions	No data were excluded.
Replication	Each experiment was repeated at least once.
Randomization	Mice were randomized to control or vaccine groups.
Blinding	The mouse survival studies were performed in a blinded fashion. The technicians who performed health checks and euthanasia of terminally-ill animals were blinded to the identity of the groups as the cages were coded.

Reporting for specific materials, systems and methods

We require information from authors about some types of materials, experimental systems and methods used in many studies. Here, indicate whether each material, system or method listed is relevant to your study. If you are not sure if a list item applies to your research, read the appropriate section before selecting a response.

Materials & experimental systems

n/a	Involved in the study	
☐	☒	Antibodies
☐	☒	Eukaryotic cell lines
☒	☐	Palaeontology and archaeology
☐	☒	Animals and other organisms
☒	☐	Human research participants
☒	☐	Clinical data
☒	☐	Dual use research of concern

Methods

n/a	Involved in the study	
☒	☐	ChIP-seq
☐	☒	Flow cytometry
☒	☐	MRI-based neuroimaging

Antibodies

Antibodies used	Fluorescently-labeled antibodies for analysis by flow cytometry were: CD3-BV605 (BD Biosciences; clone 1702), CD4-BV510 (BD Biosciences; clone M4-5), CD8-PE-Cy7 (eBioscience; clone 3–6.7), CD44-eF450 (eBioscience; clone M7) and a T cell lineage negative redFluor710-labeled antibody cocktail consisting of the following markers: B220 clone A3-6B2, CD11b clone 1/70, CD11c clone 418, CD19 clone D3, F4/80 clone BM8.1 (Tonbo Biosciences, San Diego, CA, USA). Dendritic cells were stained with anti-CD11c (PE-Cy7, eBioscience), anti-CD40 (APC, eBioscience) and anti-CD80 (PerCP-Cy5.5, BD Biosciences) then measured by flow cytometry. Detection by IgG ELISAs was performed using AKP-conjugated rabbit anti- mouse IgG (Sigma) as a secondary antibody.
Validation	Antibodies were validated prior to use based on the conditions suggested by the manufacturer

Eukaryotic cell lines

Policy information about cell lines

Cell line source(s)	ATCC
Authentication	the cell lines were not authenticated b/c they were ordered and used directly from ATCC.
Mycoplasma contamination	Cell lines tested negative for mycoplasma contamination.
Commonly misidentified lines (See ICLAC register)	not applicable

Animals and other organisms

Policy information about studies involving animals; ARRIVE guidelines recommended for reporting animal research

Laboratory animals	Male and female C57BL/6 mice, 8 to 10 weeks old, were purchased from Charles River Laboratories (Wilmington, MA)
Wild animals	not applicable
Field-collected samples	not applicable
Ethics oversight	This study was performed in strict accordance with the Guide for the Care and Use of Laboratory Animals of the National Institutes of Health (NIH). The protocols were approved by the Tulane University Institutional Animal Care and Use Committee.

Note that full information on the approval of the study protocol must also be provided in the manuscript.

Flow Cytometry

Plots

Confirm that:

☒ The axis labels state the marker and fluorochrome used (e.g. CD4-FITC).

☒ The axis scales are clearly visible. Include numbers along axes only for bottom left plot of group (a 'group' is an analysis of identical markers).

☒ All plots are contour plots with outliers or pseudocolor plots.

☒ A numerical value for number of cells or percentage (with statistics) is provided.

Methodology

Sample preparation	Single cell suspensions were prepared by homogenizing spleens on a 70 μm nylon cell strainer (Fisher) with a rubber syringe plunger from a 5 mL syringe (Fisher). The cell suspension was centrifuged at 460× g for 10 min at 4 °C. Supernatant was decanted and the cells were resuspended in 2 mL ACK red blood cell lysis buffer (Invitrogen, Waltham, MA, USA) and incubated at room temperature for 3 min and the reaction stopped with 20 mL of RMPI (Gibco, Waltham, MA, USA) containing 10% fetal bovine serum (FBS, Atlanta Biologicals, GA, USA), hereafter referred to as 10% RPMI. Cells were then centrifuged at 300× g for 10 min, supernatant was decanted, and the cells were resuspended in 5 mL 10% RPMI. The viable cells were counted on a Cellometer (Nexcelom Bioscience) using Trypan Blue (Sigma) and corrected to a final volume of 1 × 107 cells/mL from which 1 × 106 cells were added per well to a 96 well round bottom plate.
Instrument	LSR Fortessa (BD Biosciences)
Software	FlowJo (Treestar, Ashland, OR, USA).
Cell population abundance	For in vivo experiments, we sought to collect at least 1 million cells per sample. For in vitro experiments, we sought to collect at least 100,000 cells per sample. For each sample, the numbers next to gates shown in each flow plot reflect the relative abundance of the gated population within that plot. For example, in Supplemental Figure 1, in the plot showing FSC-A by lineage negative (Dump), the abundance of the Dump negative cells is 38.9% of all cells in that plot (and by extension, the Dump positive cells represent 61.1% of the cells in that plot (100% - 38.9%).
Gating strategy	Supplementary Figures 2-4 describe the gating strategy for each cell subset analyzed in the manuscript.

☒ Tick this box to confirm that a figure exemplifying the gating strategy is provided in the Supplementary Information.
